# Peptides designed from a bacteriophage capsid protein function as synthetic transcription repressors

**DOI:** 10.1016/j.jbc.2023.105373

**Published:** 2023-10-20

**Authors:** Pankaj V. Sharma, Sriyans Jain, Ranjan Sen

**Affiliations:** 1 Laboratory of Transcription, Center for DNA Fingerprinting and Diagnostics, Hyderabad, India; 2 Graduate Studies, Manipal Academy of Higher Education, Manipal, Karnataka, India

**Keywords:** Psu, peptides, transcription repressor, peptide-nucleic acid interaction, gel-shift assays

## Abstract

The bacteriophage capsid protein, Psu (polarity suppression), inhibits the bacterial transcription terminator, Rho. In an effort to find nontraditional antibacterial agents, we previously designed peptides from the Psu C terminus that function as inhibitors of Rho. Here, we demonstrated that these peptides have positive surface-charge densities, and they downregulate many genes in *Escherichia coli*. We hypothesized that these peptides could bind to nucleic acids and repress gene expression. One of these peptides, peptide 33, represses *in vitro* transcription from the *T7A1* and *P*_*lac*_ promoters efficiently by blocking the access of RNA polymerase to the promoter, a mode of transcription repression akin to many bacterial repressors. *In vivo*, expressions of the peptides reduce the total RNA level as well as transcription from *P*_*lac*_ and *P*_*osm*_ promoters significantly. However, they are less efficient in repressing transcription from the *rRNA* promoters with a very high turnover of RNA polymerase. The peptide 33 binds to both single and dsDNA as well as to RNA with dissociation constants ranging from 1 to 5 μM exhibiting preferences for the single-stranded DNA and RNAs. These interactions are salt-resistant and not sequence-specific. Interactions with dsDNA are entropy-driven, while it is enthalpy-driven for the ssDNA. This mode of interaction with nucleic acids is similar to many nonspecific ssDNA-binding proteins. Expression of peptide 33 induces cell elongation and impaired cell division, possibly due to the dislodging of the DNA-binding proteins. Overall, we surmised that these synthetic transcription repressors would function like bacterial nucleoid-associated proteins.

Antimicrobial resistance of pathogenic microbes against the majority of antibiotics is a major concern to human health. There is an urgent need to develop novel antibiotics and other antimicrobial agents to overcome the threat of antimicrobial resistance. The recent antimicrobial guideline report from World Health Organization stated bacteriophages (or phages) and proteins expressed by them as nontraditional antibacterial agents (1). Many phage proteins have specific targets in their respective host bacteria and contribute immensely to bacterial killing. These phage proteins could function as platforms for designing new antimicrobial peptides (AMPs). Earlier such efforts were made using the phage-derived endolysins, LysAB2 (2) and Pfly307 (3).

Psu (polarity suppression) is a capsid protein of an *Escherichia coli* phage P4. Psu also moonlights as an inhibitor of Rho (4, 5). It forms a V-shaped dimeric structure and binds to the exit channel of the transcription terminator, Rho (6), hindering the latter’s mRNA-translocation activity that allows it to catch up with the transcription elongation complex (7, 8). Psu’s solvent-exposed C-terminal domain (CTD) (helix 6 and helix 7) interacts directly with Rho, and its N-terminal domain stabilizes its structure (9). We cloned the C-terminal helix-7 in a suitable expressing vector and randomly mutagenized it to select clones that inhibit the functions of the Rho protein ((10), Fig. S1*A*). Two of these clones expressing 45-mer peptides, peptide 16 and peptide 33, were characterized for their specific functions to inhibit Rho-dependent termination *in vivo* and *in vitro* by direct binding to the Rho protein ((10), Fig. S1, *B* and *C*). Their expression also caused toxicity to *E. coli, Mycobacterium smegmatis*, and *Mycobacterium bovis* (10). These peptides could be used to further develop AMPs.

Now, we report that these peptides with positive surface-charge densities are also capable of downregulating a significant number of genes in *E. coli*. Peptide 33, represses *in vitro* transcription by blocking the access of RNA polymerase (RNAP) to the promoter sequence that is akin to many bacterial repressors. *In vivo*, expression of the peptides 33 reduces the total RNA level as well as transcription from specific promoters significantly. The peptide 33 binds to both ssDNA and dsDNA as well as to RNA exhibiting preferences for the single-stranded DNA and RNAs. The mode of their interaction with nucleic acids is similar to many ssDNA-binding proteins. Expression of peptide 33 induces cell-division impairment which eventually leads to cytotoxic effects. We surmised that their ability to function both as synthetic transcription repressors and Rho-inhibitors qualify these as ideal lead compounds for designing antimicrobial agents.

## Results

### Peptide-induced downregulation of genes

**Figure 1:**
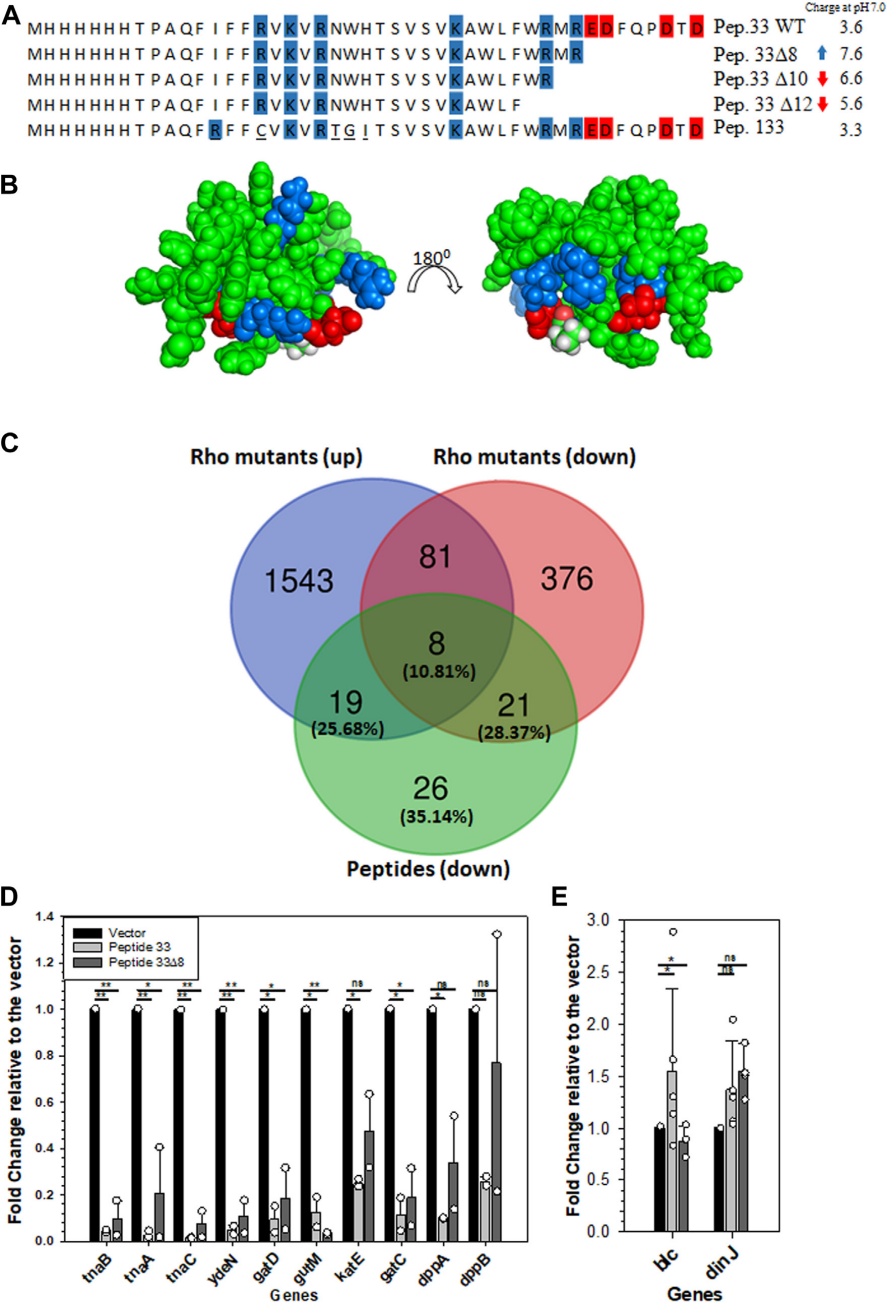
**Downregulated genes by the peptides and surface charge density of peptide 33.***A*, charged amino acids are highlighted on the amino acid sequences of the peptides. *Blue* and *red* colors represent positively and negatively charged amino acids, respectively. Net charges at pH 7.0 were calculated using online tools (https://protcalc.sourceforge.net and Expasy’s Compute pI/Mw tool). *B*, surface charge densities are shown on the space-filled models of peptide 33. R16, K18, R20, K29, R35, and R37 are colored *blue* while E38, D39, D43, and D45 *green* are in *red*. *C*, Venn diagrams showing the total number of upregulated and downregulated genes in the presence of the Rho mutants (N340S, G324D, Y80C, G51V, and P279S) or cells treated with bicyclomycin or when Psu is expressed and downregulated genes in the presence of the peptides (peptide 33 and peptide 33Δ8) obtained from microarray experiments (10). These downregulated genes are only affected in the presence of peptides. Microarray data of the Rho mutants were compared with those obtained in the presence of peptide 33 and peptide 33Δ8, and genes were selected that are neither upregulated nor downregulated in the presence of the Rho mutants. *D*, bar diagram showing the RT-qPCR data of the indicated genes in the presence of the peptides relative to that obtained in their absence in the cells (vector only). *E*, bar diagram showing the RT-qPCR data of the indicated genes that were not affected by the expression of peptides. The error bars in the graph represent SD obtained from 3 to 5 independent measurements. The color of the bars represents the same experimental conditions as in *E*. The ANOVA statistical analysis parameters are indicated as, ns = not significant, ∗*p* ≤ 0.05, ∗∗*p* ≤ 0.01, ∗∗∗*p* ≤ 0.001, and ∗∗∗∗*p* ≤ 0.0001. Psu, polarity suppression; RT-qPCR, reverse transcription-quantitative PCR.

The 45-mer peptide 33 consists of six positively charged amino acids and four negatively charged amino acids with a net charge of +3.6 (at pH 7.0) and a pI of 10 (Fig. 1*A*), and they are surface exposed and capable of forming ionic interactions at neutral pH (Fig. 1*B*). Earlier, we observed that up to eight amino acid deletions (peptide 33Δ8) from the CTD of peptide 33 did not affect its Rho-binding or cell growth inhibitory functions (10). The net charge of peptide 33 changes significantly upon CTD deletions, which in turn affects their functions. We hypothesized that their positive charge densities could allow them to bind to nucleic acids, which would affect gene expression. It should be noted that in the deletion derivatives, the positive charge densities remain either the same or moderately reduced even though the net charges are different. Hence, in addition to the role of the net positive charge density of peptide 33 to bind nucleic acid, it is possible that there exist sequence-specific interactions between amino acids of the peptide and the nucleic acid bases *via* H-bonding. So, we also studied the effects of expression of peptide 133 which has different amino acids in four positions but has a similar net charge relative to the full-length peptide 33 (Fig. 1*A*, amino acid variations are underlined).

Earlier (10), we have described detailed microarray profiles of the gene expression patterns of MG1655 strain expressing peptide 33 and peptide 33Δ8 (an 8 amino acid deletion from CTD), and compared the pattern of upregulated genes with those obtained in the presence of various Rho mutants. However, a significant number of genes were also observed to be downregulated upon expressions of these peptides. We compared these downregulated genes with both the upregulated and downregulated genes that are common when the same MG1655 strain expresses different Rho mutants (N340S, G324D, Y80C, G51V, P279S (11, 12, 13, 14); or treated with Rho inhibitor, bicyclomycin, or when Psu is expressed (Fig. 1*C*; (11, 12, 13, 14)). We observed that 74 genes were downregulated more than 2-fold when the peptides were expressed and out of them 35.14% were unique to the peptides and were not common to those up or downregulated in the presence of the Rho mutants (Fig. 1*C*). Nineteen genes are upregulated in the Rho mutants but are downregulated in the presence of the peptides (Fig. 1*C*). Most of the genes that are downregulated specifically in the presence of the peptides belong to various metabolic pathways (Fig. S2*A*). It should be noted that the number of peptide-induced downregulated genes is underestimated. Apart from the 26 unique (35.14%) genes, there must be more genes affected by the peptide among the 74 genes that are downregulated in the presence of Rho mutants. Also, this analysis only estimated the number of promoters that are available for the peptides to bind during the log phase culture. There could be many more promoters that are affected by these peptides.

We validated the microarray data of a few of these downregulated genes by reverse transcription-quantitative PCR (RT-qPCR) (Fig. 1*D*). Expressions of both peptide 33 and peptide 33Δ8 reduced the expression levels of the majority of these genes by 4- to 5-fold. It should be noted that two genes, *blc*, and *dinJ* were not downregulated in the presence of the peptides (Fig. 1*E*). This indicates that these peptides repress gene expressions of a specific set of genes, which led us to further hypothesize that these peptides might function as transcription repressors.

### Inhibition of *in vitro* transcription by peptide 33

**Figure 2:**
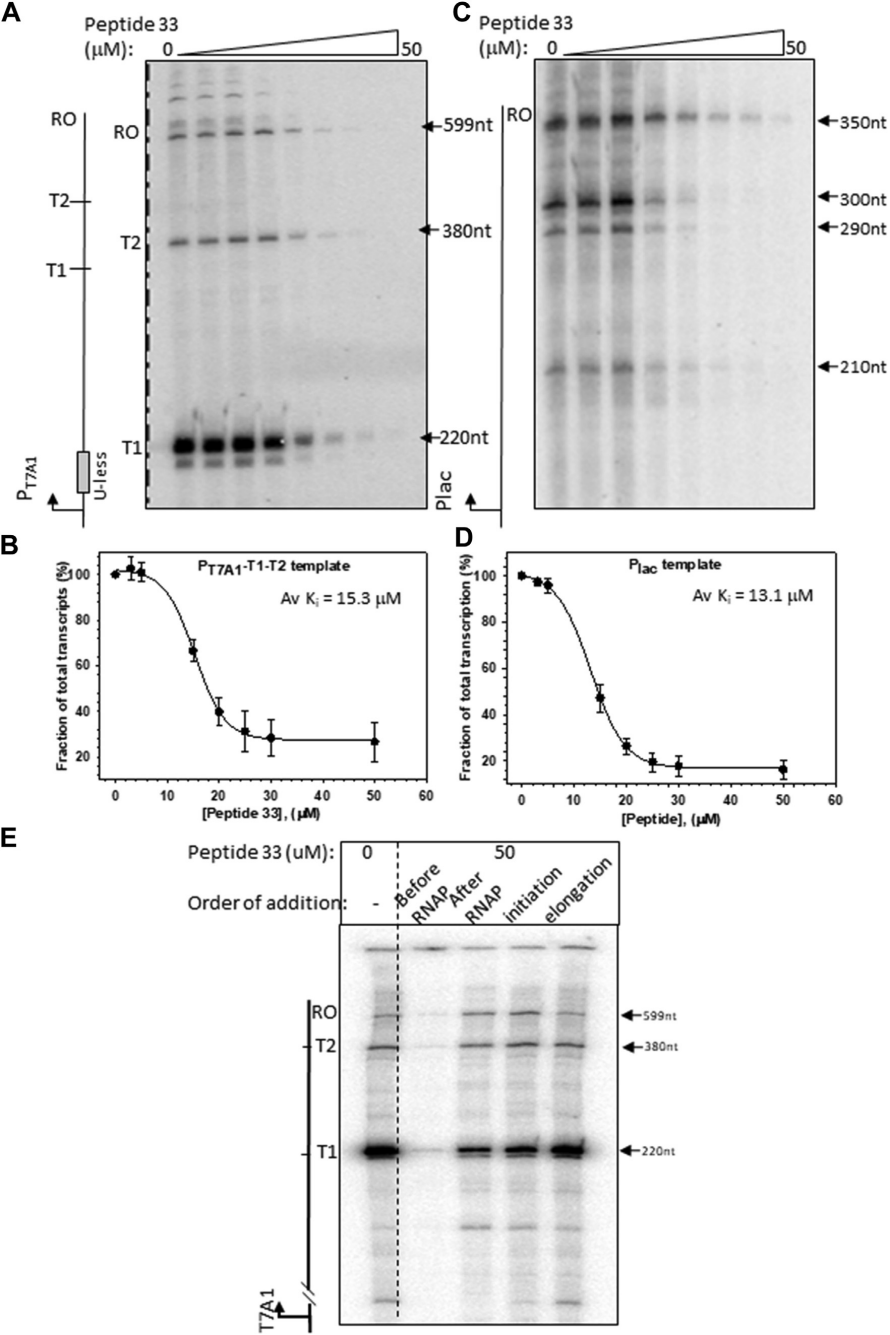
**Repression of *in vitro* transcription by peptide 33.***A*, autoradiogram showing the *in vitro* transcripts in the presence of increasing concentrations of peptide 33 as indicated. The DNA template used in this experiment is shown next to the autoradiogram. On this template, the majority of the transcripts originating from the T7A1 promoter end at T1 and T2 terminators, and a small fraction reached the end of the template (RO). This template has an initial sequence that codes a U-less sequence stretch after the transcription start site so that in the absence of UTP, a 23-mer initiation complex will form if a dinucleotide ApU is provided. *B*, plot showing the fraction of total transcripts (T1 + T2 + RO) in the presence of increasing concentrations of the peptide 33 relatives to that obtained in its absence. The average inhibition constant (*K*_*i*_) was obtained from the average concentration of the peptide required to reduce 50% of the total transcripts. Error bars and the average *K*_*i*_ were obtained from three independent measurements. *C*, autoradiogram showing the *in vitro* transcription using a terminator-less template where transcription is initiated from the *P*_*lac*_ promoter. Major transcripts obtained from the transcriptions reached the end of the template (RO) in the presence of increasing concentrations of peptide 33 (RO). *D*, plot showing a decrease of transcripts from *P*_*lac*_ promoter in the presence of increasing concentrations of peptide 33. Calculation of average Ki was done in the same way as in (*B*). *E*, effect of peptide 33 upon addition at different stages of transcription. Peptide 33 was added to the reaction before the addition of RNAP (before RNAP), after the addition of RNAP (after RNAP), after the formation of EC_23_ complex (initiation), and during the chasing of EC_23_ complex (elongation). EC, elongation complex; RNAP, RNA polymerase.

To explore the molecular basis of peptide-induced downregulation of genes, we performed *in vitro* transcription assays using purified *E. coli* RNAP and DNA templates where transcription was directed from the *T7A1* (Fig. 2, *A* and *B*) and *lac* promoters (Fig. 2, *C* and *D*) in the presence of increasing concentrations of peptide 33. These two promoters are model promoters and are used in *in vitro* transcription assays since these are very strong generating high transcription signals under *in vitro* conditions. Transcription initiating from the T7A1 promoters terminates primarily at the two rho-independent terminators, T1 and T2, fused downstream of the promoter. For the transcription initiating from the *lac* promoter transcription terminates at the end of the template (run-off [RO]). Increasing concentrations of the peptide decreased the total amounts of transcription ([T1 + T2 + RO] for the T7A1 promoter, and RO for the Lac promoter). Plots of the fractions of all the transcripts against the concentrations of peptide 33 revealed an inhibition constant in the range of ∼13 to 15 μM (Fig. 2, *B* and *D*), which indicates that this peptide inhibited transcription from these two promoters efficiently.

**Figure 3:**
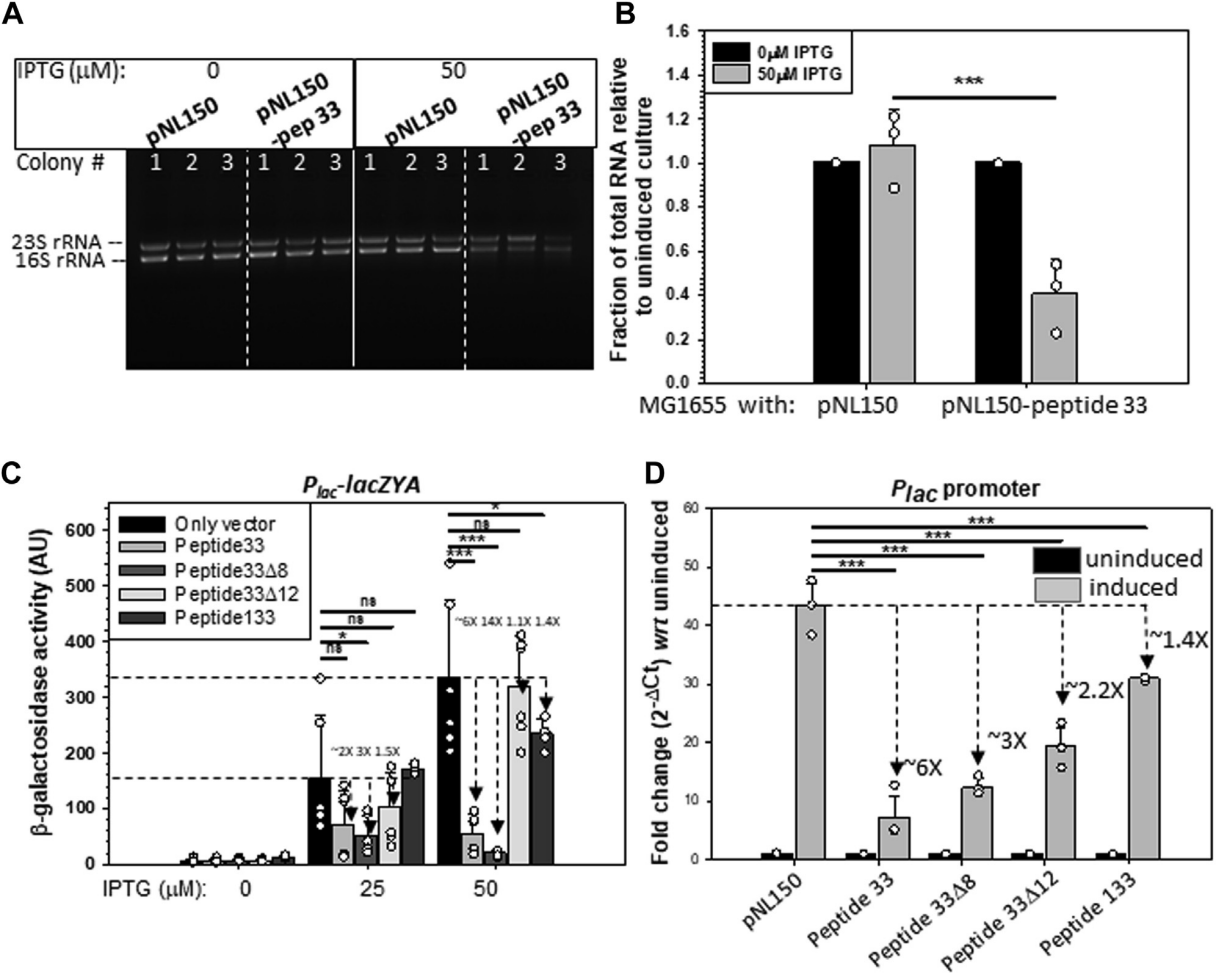
**Repression of *in vivo* transcription by the peptides**. *A*, ethidium bromide stained agarose gel showing the total RNA extracted from the MG1655 strains transformed with indicated plasmids, and the resultant strains were grown either in the absence (0 IPTG) or the presence (50 μM) of the inducer, IPTG. Total RNA extracts are dominated by the 23s and 16s rRNAs. *B*, bar diagram showing the fractions of the rRNA produced upon induction of pNL150 and pNL150-peptide 33 vectors relative to that were obtained in the absence of the inducer. Errors were obtained from three independent measurements. *C*, bar diagrams showing the β-galactosidase activities from the *lacZYA* operon fused to the *P*_*lac*_ promoter from the strains that are transformed with the plasmids, pNL150 expressing peptide 33, peptide 33Δ8, peptide 33Δ12 and peptide 133 together with an empty pNL150 grown in the presence of indicated concentrations of IPTG. Error bars were obtained from 3 to 5 independent measurements. *D*, bar diagrams showing the *in vivo* transcription activities of *P*_*lac*_ promoter obtained from RT-qPCR measurements in the presence of different peptides as indicated. Fold-change values from the RT-qPCR data were obtained relative to the uninduced ones. Reductions of fold change induced by each peptide are indicated. The error bars in the graph represent SD and were obtained from 3 to 5 independent measurements. The ANOVA statistical analysis parameters are indicated as, ns = not significant, ∗*p* ≤ 0.05, ∗∗*p* ≤ 0.01, ∗∗∗*p* ≤ 0.001, and ∗∗∗∗*p* ≤ 0.0001. RT-qPCR, reverse transcription-quantitative PCR.

**Figure 4:**
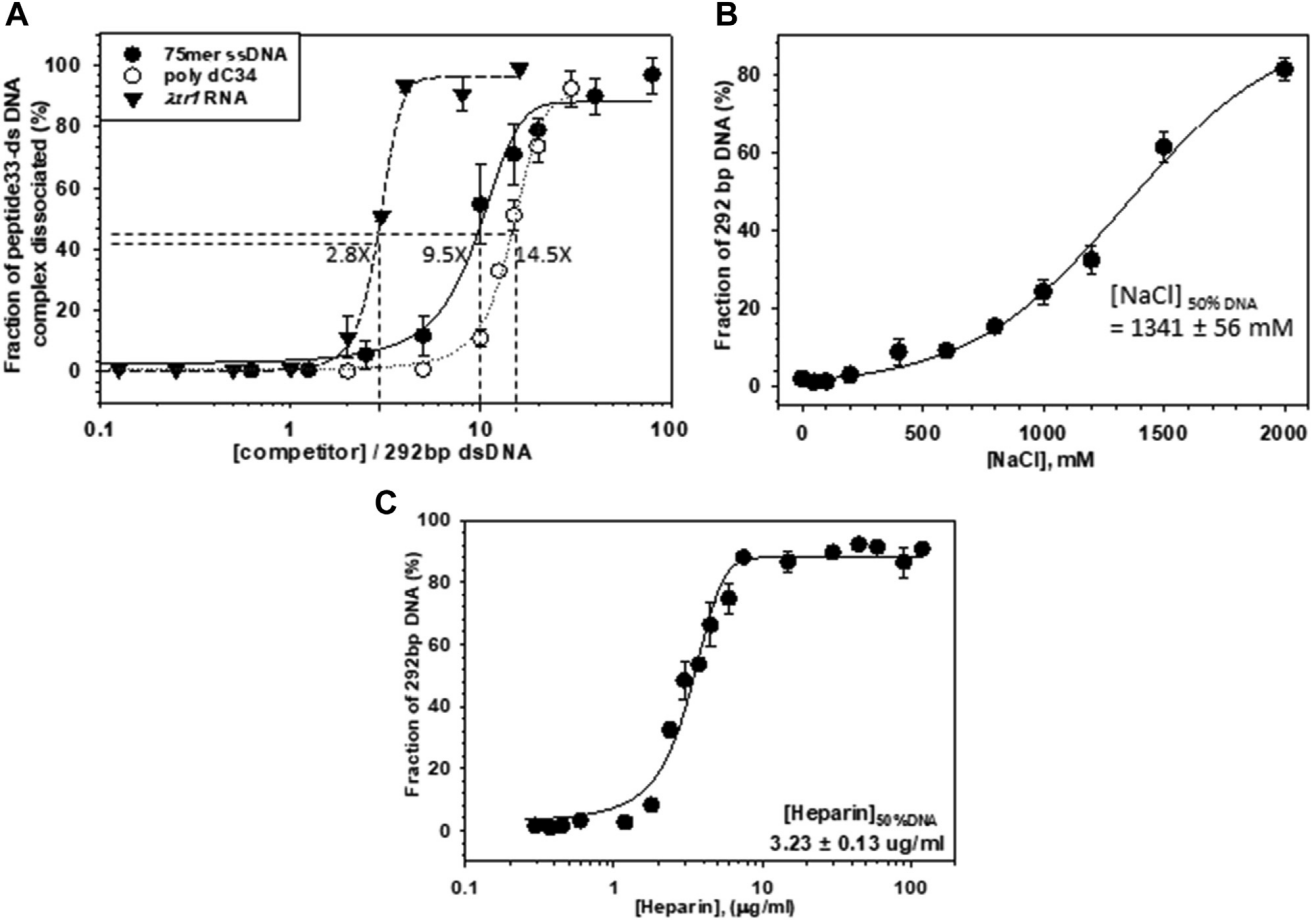
**Nucleic acids binding properties of the peptide 33**. *A*, sigmoidal plots showing the dissociation of peptide 33-dsDNA complex in the presence of increasing concentrations of the competitors, 75 mer ssDNA, poly dC_34,_ and *λt*_*R1*_ RNA. Amounts of competitors required (expressed as fold-excess over dsDNA concentration) for 50% dissociation of the peptide 33-dsDNA complex are indicated. Ratios of concentrations of the competitors and the 292 bp DNA are plotted on the X-axis. Error bars were obtained from three independent measurements. The indicated DNA and RNA concentrations were measured in terms of the whole molecule. Sigmoidal plots show the dissociation of the same complex in the presence of increasing concentrations of NaCl (*B*) and heparin (*C*). Concentrations of each of these two chemicals are required to dissociate 50% of the peptide are indicated. Errors (SD) were obtained from three measurements.

Next, we checked at which stage of transcription this peptide inhibits transcription (Fig. 2*E*). We added peptide 33 to the DNA template (described in Fig. 2*A*) before adding the RNAP (before RNAP lane), added after the RNAP-promoter binary complex was formed (after RNAP lane), added to the elongation complex (EC_23_) initiation complex (initiation lane), and during the transcription elongation reaction of EC_23_ complex (elongation lane). On this template (see the cartoon in Fig. 2*A* adjacent to the autoradiogram), the initial sequence codes for a U-less stretch so if the transcription is initiated with an ApU in the absence of UTP, a 23-mer initiation complex EC_23_ is formed. We observed that the transcription process was most significantly inhibited when peptides were added to the DNA template in the absence of any other components (before RNAP lane). There was a moderate reduction in transcription when it was added to the RNAP-promoter binary complex, and no effect of the peptide was observed in the later stages of transcription. As the peptide 33 effects are limited to the DNA binding as well as RNAP-promoter binary complex formation steps, most likely there would be no effect on the abortive transcription step(s), a postinitiation activity of the RNAP. This indicates that the presence of peptide 33 blocks the access of the RNAP for the promoter, and functions as a classical transcription repressor. RNAP-promoter binary complex on the T7A1 promoter usually has a high dissociation rate (15), so peptide 33 binding to DNA could have partially competed with this complex (lane after RNAP).

### Inhibition of *in vivo* transcription by the peptides

**Figure 5:**
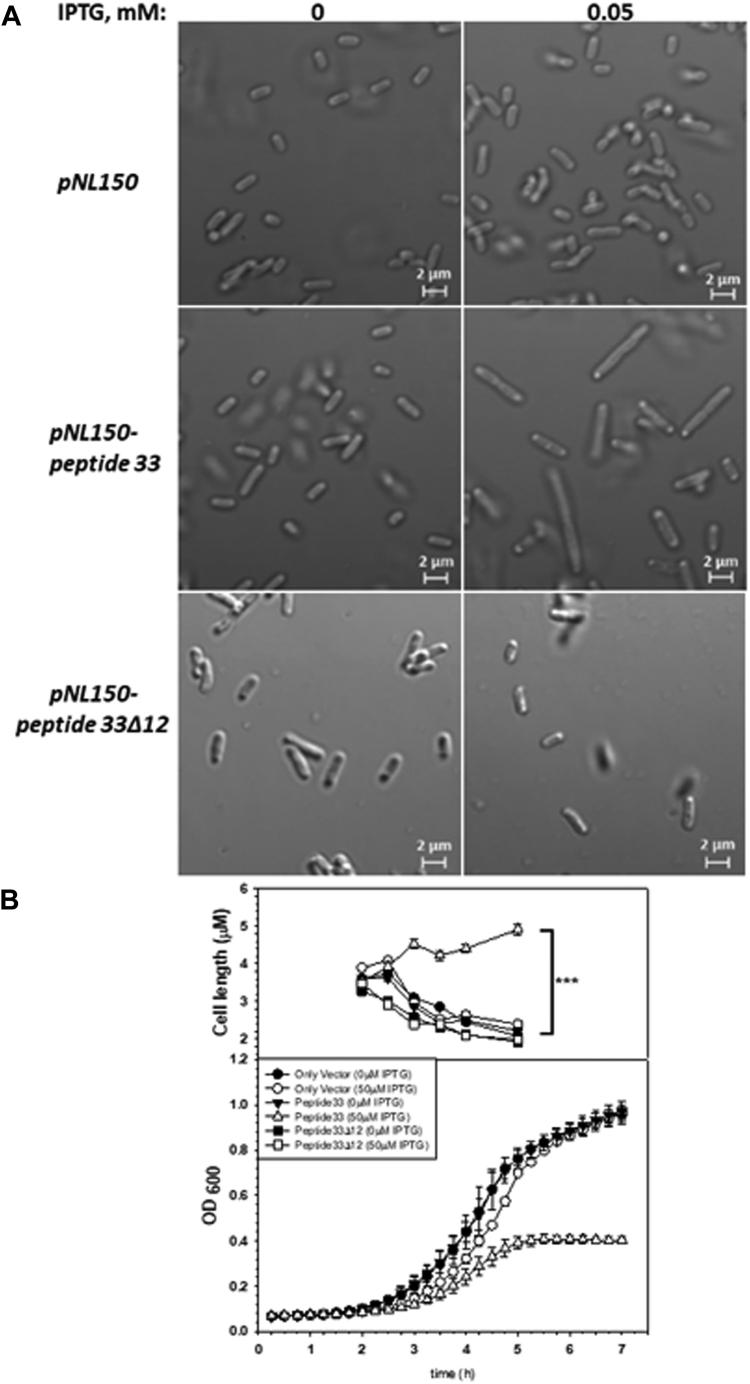
**Morphological changes in the *Escherichia coli* cells treated with peptide 33**. *A*, confocal microscope DIC images of cells after 5-h expression of peptide 33 and those carrying the only vector. *B*, growth curve of *E. coli* MG1655 cells with and without peptide 33. The *upper panel* showed plots of the average length of cells either expressing peptide 33 (in the presence or absence of IPTG) or peptide 33Δ12 or those carrying only the vector at 6 time points of growth curves (2, 2.5, 3, 3.5, 4, and 5 h). Each point represents the average size ± SD of at least 200 cells (∗∗∗*p* ≤ 0.001). DIC, differential interference contrast.

Next, we measured the total RNA level from a mid-log phase culture of the *E. coli* MG1655 strain in the presence or absence of the expressions of peptide 33. The peptide 33 was expressed from the pNL150 expression vector inducible by IPTG. The strain was transformed either with the vector or with the pNL150 expressing peptide 33, and cells were grown up to the mid-log phase either in the presence or absence of the inducer, IPTG. The strains expressing peptide 33 took a long time to reach the mid-log phase as it caused growth defects (Fig. 5*B*). The *in vivo* level of peptide 33 was significantly high under this condition as estimated from its mRNA level (Fig. S2*B*). Figure 3*A* shows the amount of total extracted RNA from the mid-log phase culture. The total RNA in ethidium–stained agarose gel is usually dominated by the 23S and 16S RNAs. About 60% reduction of the level of the total RNA was observed upon *in vivo* expression of peptide 33 in the presence of IPTG (Fig. 3*B*). This indicates that peptide 33 expression affects the *in vivo* transcription process, and this is consistent with the fact that the peptides induce growth defects in bacterial strains (10).

To explore the peptide-induced repression of promoter activities *in vivo*, we chose the very well-studied *lac* promoter. We used the MC4100 (*lac*^*-*^) strain having a *P*_*lac*_*-lacZYA* fusion as a lysogen in its chromosome. This strain was transformed with a pNL150 vector expressing peptide 33, peptide 33Δ8, peptide 33Δ12, and peptide 133. The β-galactosidase activities obtained from the expression of this fusion were measured in the absence and presence of the inducer, IPTG (Fig. 3*C*). It should be noted that IPTG, in addition to inducing the expressions of the peptides also induces the *P*_*lac*_ promoter which was evident from the very high level of activity of this enzyme in the strain having only the pNL150 vector (black bars). We observed that peptides 33 and 33Δ8 induced reduction of β-galactosidase activities by 6 and 14-folds, respectively, at the highest concentration of IPTG. The deletion derivatives, peptide 33Δ12, as well as the peptide 133 having amino acid changes in four positions, were less effective in repressing this activity, which is consistent with their inability or reduced abilities to inhibit the growth of *E. coli* (10).

The reduction in β-galactosidase activities could arise from peptide-induced translation defects. So, we directly measured the quantity of transcript made from the *P*_*lac*_ promoter in the presence or absence of the peptides by RT-qPCR (Fig. 3*D*). Upon expressions of the peptides, 1.4- to 6-fold reductions of the levels of *P*_*lac*_ transcripts were observed, which indicates that the reduction of β-galactosidase activities was due to the repression of transcription from this promoter. The least amount of reduction in transcripts was observed in the presence of peptide 133. We performed similar RT-qPCR assays with two other promoters, *P*_*rrnA*_ and *P*_*osmE*_ to check the repressions by the peptides from other promoters. The repression was moderate in these cases with a 2- to 3-fold reduction in transcript levels (Fig. S3). These results suggest that peptide 33 and peptide 33Δ8 functions as transcription repressors *in vivo*. However, their repression efficiency varies among different promoters. Likely, the promoters with a faster turnover rate (like *rRNA* promoter) could be less repressed by these peptides as these promoters are unoccupied by RNAP for a lesser time duration. The *in vivo* promoter repressor function is a specific property of peptide 33 as this function was significantly reduced when its deletion derivative (peptide 33Δ12), as well as a derivative having multiple amino acid changes (peptide 133), were present.

### Peptide-nucleic acids interaction

The above results indicated that peptide 33 directly binds to DNA to inflict transcription repression. Hence, we explored its mode of direct interactions with nucleic acids. We have chosen radiolabeled-dsDNA (160 bp, 292 bp, and 499 bp), ssDNA (26-mer, 34-mer, and 75-mer), and RNA (283 nt *gfc* RNA and 25-mer polyC) of different sizes and sequences, and employed gel-shift assays with peptide 33 to determine its affinity for them (Fig. S4, *A* and *B* show representative gel-shift assays). All the binding isotherms followed sigmoidal curves indicating multiple cooperative binding of the peptide on these templates (Fig. S4, *A*–*C*). Table 1 summarizes the dissociation constants of these interactions. ssDNA and RNA have 3- to 4-fold higher affinities than dsDNA. Shorter ssDNA or RNA molecules have lesser affinity than their lengthier single-stranded counterparts, which could be due to the presence of fewer binding sites resulting in lesser cooperativity in binding (Fig. 4*A*). We concluded that peptide 33 binds nucleic acid sequence nonspecifically at multiple sites along the templates and prefers ssDNA as well as RNA over dsDNA.

**Table 1:** Dissociation constants of peptide-DNA/RNA interaction

ssDNA/dsDNA/ssRNA	*K*_*d*_ (μM)
26-mer ssDNA	2.0 ± 0.170
34-mer polydC	1.0 ± 0.034
75-mer ssDNA	0.74 ± 0.042
160 bp dsDNA	4.0 ± 0.063
292 bp dsDNA	2.75 ± 0.093
499 bp dsDNA	4.7 ± 0.137
283 nt *gfc* RNA	1 ± 0.03
25-mer polyrC	1.9 ± 0.12

Dissociation constants (*K*_*d*_) were measured from gel-shift assays. SEM was obtained from three measurements. Fractions of the bound complex were plotted against the concentrations of peptide 33 and the data were fitted to a sigmoidal binding isotherm to determine these values.

Next, we measured the thermodynamics of peptide 33–DNA interactions by measuring the binding constants at different temperatures using fluorescence spectroscopy. We used different dsDNA and ssDNA molecules labeled with the fluorescent probe fluorescein at their 5′end. The titration of DNA with increasing concentrations of peptide 33 resulted in gradual quenching of the fluorescence signal of the DNA (Fig. S5*A*). The dissociation constant (*K*_*d*_) was calculated by plotting the normalized fluorescence intensity at 520 nm against the peptide concentrations and fitting the curve to the hyperbolic decay model (Fig. S5*B*). To examine the mode of interactions between DNAs with peptides, we determined the thermodynamic parameters. The dissociation constants for DNA–peptide 33 interaction were determined at multiple temperatures and were fed to the Van’t Hoff equation. The thermodynamic parameters were determined from Van’t Hoff plots obtained by plotting apparent binding constants (*K*_*b*_) against the temperature (Fig. S5). The high negative values of free energy change (ΔG) indicated spontaneous and stable interactions between DNA and peptide 33 (Table 2). The interactions of peptide 33 with dsDNAs were entropically driven and enthalpically unfavorable as indicated by the positive values of enthalpy changes (ΔH) and entropy changes (ΔS). The positive enthalpy change indicates endothermic binding, which might have arisen from the interactions of positive residues of the peptide and phosphate backbone of DNA strands (16). The major contribution of entropy came from the release of ions and water molecules from the complimentary surfaces, which is characteristic of the nonspecific interactions of DNA–protein complexes (16, 17). In contrast to the interactions with dsDNAs, the peptide interaction with the ssDNA resulted in a large negative enthalpy change and a small negative entropy change (Table 2) indicating that the peptide 33–ssDNA interactions are exothermic and are enthalpically driven. This difference in the binding modes of ssDNA and dsDNA to peptide could be attributed to the relatively flexible structure of ssDNA as compared to the more rigid conformations of dsDNA. Further, the solvent-exposed bases of ssDNA, unlike dsDNA, can interact through π-π stacking with the aromatic groups of the peptide residues contributing to the increased enthalpy change and reduced entropy change (18). This further validates our observation that the interaction between peptide 33 with ssDNA is more stable than that with dsDNA.

**Table 2:** Thermodynamic parameters for DNA–peptide 33 interaction

DNA	ΔH (kJ⋅mol^−1^)	ΔS (kJ⋅mol^−1^⋅K^−1^)	ΔG (kJ⋅mol^−1^) at 25 °C
100 bp dsDNA	31.689	0.224	−35.078
160 bp dsDNA	20.041	0.191	−36.818
75-mer ssDNA	−37.341	−0.0096	−34.488

Values were obtained from the Van’t Hoff plots shown in Fig. S5.

We then investigated the ability of different RNA and DNA molecules of different lengths to compete with the 292 bp DNA–peptide 33 complex. We preformed a radiolabeled 292bp-dsDNA-peptide 33 complex and added increasing concentrations of different competitors,75-mer ssDNA, poly dC_34_ (34-mer), and 284 nt *λT*_*R1*_ RNA. We observed that *λT*_*R1*_ RNA is more preferred by peptide as the dsDNA-peptide complex as it was efficiently released by RNA as compared to both ssDNA. A total of 2.8 times excess RNA was required to release 50% of 5’ [P^32^] labeled dsDNA from the dsDNA-peptide complex, while 9.5 and 14.5 times excesses of 75mer ssDNA and poly dC_34_ were required to release 50% dsDNA (Fig. 4*A*). This indicates that longer stretches of RNA offer more binding sites for the peptide, which is consistent with the binding data described in Table 1.

The DNA–peptide complex interaction was quite stable and resistant to high salt concentrations (Fig. 4*B*). The 50% dissociation of the complex required ∼1.3 M NaCl. The dsDNA-peptide complex was dissociated when negatively charged heparin was added with a dissociation constant (*K*_*d*_) of 3.23ug/ml heparin (Fig. 4*C*). This suggests that polyamines like heparin could also bind to peptide 33. The salt resistance arose most likely from nonionic components, like aromatic ring-base stacking and hydrogen bonding interactions between the amino acids and the nucleic acids.

### Peptide 33 induces elongated cell shape

In addition to affecting the transcription process, by the virtues of such stable interactions with both RNA and DNA, the expression of the peptides would also impair replication and other DNA-dependent processes leading to growth defects as well as changes in morphology. So, we investigated the physiological effects of the expressions of peptide 33 *in vivo*. We observed that under the confocal microscope, the cells grown until the mid-log phase expressing this peptide were significantly longer in size compared to the cells that didn’t express or expressed a 12 amino acid deletion derivative of (peptide 33Δ12) the peptide, and the size enhancement was ∼2.5 fold (Fig. 5*A*). We then monitored the cell size changes in different growth phases. Bacterial samples were collected at different growth stages and observed under a confocal microscope. The plots of cell length against the time of growth indicated that the cell length decreased as the growth phase progressed from the early to late log phase when the cell did not express peptide 33 or expressed the peptide 33Δ12, while the cell became longer in the late growth phase when the WT peptide 33 was expressed (Fig. 5*B*). This phenomenon could be attributed to impaired cell division due to delayed replication resulting from peptide-induced repression of the gene expressions of a significant number of genes. Reduction of mRNA leads to a slow accumulation of proteins, which in turn slows down the subsequent metabolic steps. However, the DNA binding properties of the peptides could directly impair the replication process in the events when it competes with the DNA replication machinery for binding to DNA.

Many antibiotics that target DNA replication (ciprofloxacin; (19)), protein (kanamycin; (20), chloramphenicol; (21, 22), gentamycin; (23), amikacin; (21)) and RNA synthesis were also shown to produce elongated cells phenotypes. A certain level of accumulation of proteins involved in replication and cell division initiates the division of the cell (24, 25, 26). The peptide-induced global repression of transcription delayed the protein accumulation process inside the cell, which did not allow the cells to reach the biomass required to divide causing the increase in cell length in the presence of peptides.

## Discussion

Earlier, we reported the design of novel peptides from a bacteriophage capsid protein Psu that interacts with the transcription terminator protein, Rho [(10), Fig. S1]. Here, we showed that these peptides could also function as transcription repressors. We provided the following evidence. Expressions of these peptides downregulated a sizeable number of unique genes (Fig. 1, *C* and *D*). The peptide 33 reduces promoter-directed transcription by blocking the entry of RNAP to the promoter (Fig. 2). Both peptide 33 and its eight amino acid deletion derivative, peptide 33Δ8, efficiently reduced *in vivo* promoter-directed transcription as well as global RNA-level while two of its mutant derivatives had significantly reduced efficiency for this function (peptide 33Δ12 and peptide 133; Fig. 3). Peptide 33 binds very stably in a salt-resistant manner (Fig. 4) but nonspecifically to multiple sites of nucleic acids having relatively more affinity for ssRNA (Table 1) and its mode of interaction is akin to many ssDNA-binding proteins (Table 2). Their function as transcription repressors, as well as their stable binding with nucleic acids led to growth defects and cell elongation upon their *in vivo* expressions (Fig. 5). We propose that these peptides could function similarly to bacterial nucleoid-associated proteins that function as general nonspecific transcription repressors (27).

We observed that peptide 33 prefers ssRNA/ssDNA over dsDNA *in vitro* (Table 1). We believe that these peptides could preferentially target dsDNA (bacterial chromosome) over RNA *in vivo* because ssRNA stretches devoid of any other proteins are rare inside the cell as they would be degraded. The reductions in peptide-induced β−galactosidase expressions (Fig. 3) were due to the reduction of the transcription and not translation. The latter could have been reduced if the peptides targeted the mRNAs. However, these experiments are not conclusive and it is possible that the high-affinity RNA-binding ability of the peptides could come into play when free RNAs are available under certain conditions in the cell.

We have earlier shown that these peptides also inhibit the function of bacterial transcription terminator, Rho (10). Hence, at a given time, the peptides are capable of binding to both the Rho and the nucleic acids. Rho has a very high affinity binding to the *rut* (rho utilization) sites on the mRNA through its N-terminal primary binding site (PBS) (6). Peptides also bind to this same PBS of Rho (10). As the Rho-RNA binding is very stable, it is likely that peptides would not be able to bind the RNA-bound Rho as the latter has weaker affinity. We speculate that the peptide could only target the free Rho that is not engaged in the transcription termination process. However, the growth retardation, as well as cell-shape alteration effects induced by the peptide expression, could be the result of its functions both as a transcription repressor and the Rho-inhibitor. This proposition is consistent with the fact that the expressions of the peptides in the cell affect various pathways like bacterial chemotaxis, flagella assembly, exopolysaccharide biosynthesis, and so on (Fig. S6) in addition to the metabolic pathways mentioned earlier (Fig. S2*A*).

AMPs are prevalent in all forms of life. They are part of the natural defense mechanism of the organism. Pleurocidin, a 25-residue peptide derived from winter flounder’s skin secretions and its derivatives, P-der, shows a broad-spectrum antibacterial activity (28) and acts by blocking the synthesis of macromolecules. Similarly, a group of human neutrophils-derived peptides, HNP-1, HNP-2, and HNP-3 defensins also inhibit the synthesis of DNA, RNA, and proteins in *E. coli* and also cause cell death (29). Another such small peptide that has the potential to be useful against drug-resistant bacteria is indolicidin. It is a 13-mer peptide with tryptophan-rich sequences and belongs to the cathelicidin family of host defense peptides (30, 31, 32). It binds to dsDNA, effectively wrapping around the DNA molecule. In addition to these, several attempts were made to synthetically designed peptide transcription repressors (33, 34, 35, 36). However, this is the first report in which we describe peptides functioning like bacterial nucleoid-associated proteins.

The AMPssthat we described here are too long (38-mer) to be delivered to any bacteria. Even the 8 to 10 amino acid deleted ones are also too large to cross the bacterial membranes. Further, designing is required to make these peptides deliverable and specifically targeted to the bacterial DNA instead of human DNA. Alternatively, these peptides cloned in plasmids could be transferred inside the bacteria *via* conjugation methods or exploiting the properties of natural transformation competence of several pathogenic bacteria, or bacteriophages could be used as delivery vehicles.

## Experimental procedures

### Materials

NTPs were purchased from GE HealthCare. We obtained [γ-^32^P]ATP (3000 Ci/mmol) and [α-^32^P]CTP (3000 Ci/mmol) from Jonaki, BRIT. WT *E.coli* RNA polymerase holoenzyme, T4 PNK, and T4 DNA ligase were from New England Biolabs. Antibiotics, IPTG, lysozyme, DTT, and bovine serum albumin were obtained from the United States Biochemical Corporation. T7 RNA Polymerase was from Lucigen. Streptavidin-coated magnetic beads were purchased from Promega. Taq DNA polymerase was obtained from Roche Applied Science. Ni-NTA agarose beads were from Qiagen and Sigma. Peptides 33 of >98% purity were synthesized by Biotech Desk Pvt. Ltd. MicroSpin G-25 columns were purchased from Amersham Bioscience, all the unlabeled oligos and 5′ fluorescein-labeled oligos were from Eurofins Genomics India Ltd, and 1× PBS was from Gibco. Four percent paraformaldehyde solution was purchased from Chemcrux Enterprises Ltd, methanol from Qualigens, 4′,6-diamidino-2-phenylindole, glycerol, and poly-L-lysine solution (0.1 % (w/v) in H2O) was purchased from Sigma. Among the different peptides, the peptide 33 was found to be the most water soluble, so we performed all the *in vitro* experiments with it.

Details of the bacterial strains, plasmids, and oligos are described in Tables 3 and S1.

**Table 3:** Strains and plasmids used in this study

Strains	Description	Reference
BL21(DE3)	*F-omp ThsdSB*(rB-mB-) *gal* dcm(DE3)	Novagen
DH5α	Δ (*argF-lac*) U169 *supE44 hsdR17 recA1 endA1 gyrA96 thi-1 relA1*(ø80lacZΔM15)	Novagen
RS1263	*E. coli* MG1655; K-12; WT	Lab stock
XL1-Red	*endA1 gyrA96 thi-1 hsdR17 supE44 relA1 lac mutD5 mutS mutT Tn10Tet*	Stratagene
RS445	GJ3161(MC4100) *λRS88* lysogen carrying *P*_*lac*_*–lacZYA*	(14)
Plasmids		
pRS17	pTL61T with *P*_*lac*_*-nutR-tr'-T1T2-lacZYA*, amp^R^	(43)
pRS18	pTL61T with *P*_*T7A1*_*-T1T2-lacZYA; P*_*t7A1*_ cloned at EcoRI/BamHI site of RS17, amp^R^	(37)
pRS19	pTL61T with *P*_*lac*_*-H19B nutR-lacZYA*, amp^R^	(22)
pRS22	pTL61T with *P*_*T7A1*_*-nutR-tr'-T1T2-lacZYA;* nutR-tr' fragment cloned at HindIII site of RS18, amp^R^	(37)
pRS102	pTL61T with *P*_*lac*_*-trpt-lacZYA; trpt* is cloned at HindIII/BamHI sites of PK8641,amp^R^	(37)
pRS106	pTL61T with *P*_*T7A1*_*-trpt-lacZYA; P*_*t7A1*_ cloned at EcoRI/BamHI site of RS102, amp^R^	(7)
pRS431	pK8628 with Plac-lacZYA; by deleting H19B NutR between Bam HI/Hind III and a 22-nucleotide adapter made of RS205 and RS206 is inserted; Amp^R^	(14)
pRS604	pTL61T with *P*_*T7A1*_*-λtr1-T1T2-LacZ* cloned at Hind III site of pRS22; Amp^R^	(7)
pRS1675	pNL150 only vector (without insert); Cam^R^	(8)
pRS1888	pNL150 with peptide 16 (as obtained in screening); Cam^R^	(10)
pRS1889	pNL150 with Peptide 33 (as obtained in screening); Cam^R^	(10)
pRS1960	pNL150 with Peptide 33Δ8 cloned at EcoRI/HindIII; Cam^R^	(10)
pRS2048	pNL150 with peptide 33Δ10 cloned at EcoRI/HindIII; Cam^R^	(10)
pRS2049	pNL150 with peptide 33Δ12 cloned at EcoRI/HindIII; Cam^R^	(10)
pRS2297	pUA66 with *rrnA* promoter fused with *gfpmut2* (*rrnA-gfpmut2*); kan^R^	(43)

### Gel-shift assays

The binding of ssDNA/RNA/dsDNA with peptide 33 was measured by gel-shift assays using radiolabeled nucleic acids. The ssDNA templates and poly rC_25_ were 5′ end-labeled using [γ-^32^P]ATP (3000 mCi/mmole). For dsDNA templates, the upstream oligo RS58 was 5′ labeled with [γ-^32^P] ATP and used in the PCR reactions to prepare different sizes of dsDNA for the binding assays. The RNA templates were made by *in vitro* transcription initiated from the T7 φ10 promoter followed by *λT*_*R1*_ containing sequence using [α-^32^P] UTP (3000 mCi/mmole) and T7 RNA polymerase (Ambion Inc). This DNA template was made by PCR amplification using RS139/RS341 primer pair on pRS604 plasmid. RS139 contains the T7φ10 promoter that inserted this promoter sequence upstream of the *λT*_*R1*_ terminator sequence. A total of 10 nM of labeled DNA templates were used for the gel-shift assays using the binding buffer (10 mM Tris–HCl (pH 8.0) and 250 mM NaCl) with increasing concentrations of peptide 33 (0.05 μM to 15 μM). The binding reactions were incubated at 37 °C for 10 min and loaded onto either 6% native acrylamide gel for ssDNA or 4% native acrylamide for dsDNA and RNA templates. The bound and unbound fractions of the DNA or RNA templates were visualized by phosphor imager Typhoon 9200 and quantified by ImageQuant 5.2 software. Data were then fitted to sigmoidal binding isotherms to measure the binding constants (*K*_*d*_) using SigmaPlot software (https://systatsoftware.com/sigmaplot/).

### Competition assay

For competition assays, 5′ radiolabeled 292 bp dsDNA(292 bp)-peptide 33 (10 nM: 8 μM) complex was made by incubating the dsDNA and peptide 33 at 37 °C for 10 min in the binding buffer. Then this complex was challenged with different ssDNA and RNA for an additional 10 min at 37 °C in the same binding buffer. Similarly, the peptide 33-ds DNA complex was also challenged with different concentrations of NaCl and heparin. After incubations with the competitors were over the whole reaction mixtures were loaded onto 6% native acrylamide gel, and were visualized and quantified in the same way as mentioned above. Data were then fitted to sigmoidal curves to estimate the inhibition constants or the fold excess of the competitors required to exert 50% dissociation of the peptide-dsDNA complex.

### Determination of thermodynamic parameters by fluorescence spectroscopy

To determine the thermodynamic parameters of DNA–peptide 33 interaction, the dissociation constant (*K*_d_) at different temperatures (20, 25, 30, and 37 °C) for DNA–peptide 33 complex was measured with a fluorescein-labeled 75-mer ssDNA (5′ fluorescein-labeled RS2230) and fluorescein-labeled dsDNAs of length 100 bp and 160 bp, amplified from plasmid pRS106 using primer pair RS586/RS2381 and RS586/RS209, respectively. The binding assays were performed by mixing an increasing amount of peptide 33 with 10 nM fluorescein-tagged DNA in binding buffer (10 mM Tris–Cl (pH 8) and 500 mM NaCl) in a quartz cuvette. The fluorophore was excited at 470 nm, and the emission spectra were recorded in the range of 500 to 600 nm using a Hitachi F-7000 fluorescence spectrophotometer under computer averaging of transients scan mode, with a photomultiplier tube voltage of 700 V, a scan speed of 240 nm/min and emission and excitation slit widths of 5 nm. The dissociation constant (*K*_*d*_) was estimated by plotting the normalized fluorescence intensity at 520 nm (F/F_0_) against the concentration of the peptide, where F and F_0_ represent the peak fluorescence intensity after adding the peptide (F) and initial fluorescence intensity without the peptide (F_0_). The plots were fitted using SigmaPlot version 13 to the hyperbolic equation of the form; y = y_0_ + (a ∗ b)/(b + x), where b denotes the apparent dissociation constant (*K*_*d*_). Binding isotherms were obtained at different temperatures (Fig. S5, *A* and *B*). The thermodynamic parameters were evaluated from the Van’t Hoff equation (Eq. 1):

$\ln\;K_{b}=-\frac{\Delta H}{RT}+$, where K_b_ represents the association constant and was calculated as 1/*K*_*d*_ and T and R represent absolute temperature and universal gas constant, respectively. The slope and the intercept of a linear plot (Fig. S5) between ln(K_b_) and 1/T provided the values of enthalpy change (ΔH) and entropy change (ΔS), respectively. The free energy change (*ΔG*) of the DNA–peptide interaction was then calculated from the equation: *ΔG = ΔH - TΔS*

### *In vivo*, transcription repression assays

*In vivo* transcription repression assays were performed using MC4100 strain (RS445) having a *P*_*lac*_*-lacZYA* fusion present in its chromosome as an λRS45 lysogen. This strain was transformed with a pNL150 vector expressing either peptide 33 or peptide 33Δ8 or peptide 33Δ12 and also with an empty pNL150. The transformants were used to measure the β-galactosidase activities from the *lacZYA* reporter fused to the P_lac_ promoter in the presence of different concentrations of the inducer, IPTG. These strains were grown with different concentrations of IPTG until the *A*_600_ reaches ∼ 0.3 following which the β-galactosidase activity measurements were performed in a microtiter plate set-up using a Spectramax Plus plate reader as described earlier (37).

To investigate the effect of peptide 33 on global RNA expression levels, we conducted a comparative study between cells expressing pNL150 and pNL150-peptide 33. Total RNA was isolated when the *A*_600_ reached 0.3 for all cells growing in the presence or absence of IPTG using the RNeasy Plus Mini Kit (Qiagen). To determine the RNA levels, isolated RNA was loaded onto a 1.5% agarose gel, and the intensities of the 16S and 23S rRNA were measured using ImageJ software (https://imagej.net/ij/download/). The intensities of the RNA bands of the IPTG-induced cells were normalized with that of the uninduced cells, resulting in a ratio of induced to uninduced (induced/uninduced) that were plotted as bar graphs using SigmaPlot 15.0.

### *In vitro* transcription repressors assay

*In vitro*, transcription assays were performed in transcription buffer (T-buffer; 25 mM Tris–HCl, pH 8.0, 50 mM KCl, 5 mM MgCl2, 1 mM DTT, and 0.1 mg/ml bovine serum albumin) at 37 °C. Two promoters, T7A1 and Lac, were used for these assays. We used a template having T7A1 promoter fused to T1 and T2 terminators and Lac promoter fused to random sequences. The first 23 DNA sequence of the T7A1-T1-T2 template is U-less enabling one to form a 23-mer EC_23_ in the absence of UTP. *P*_*T7A1*_ -T1T2 template fragment was amplified from the plasmid pRS22 using RS58 and RK1 primer pair. *P*_*lac*_ promoter containing DNA fragment was amplified from pRS431 using the same RS58 and RK1 primer pair. An amount of 10 nM DNA was incubated with increasing concentrations of peptide 33 in a T-buffer for 10 min, following which 50 nM WT RNAP holoenzyme was added. And incubation was continued for another 10 min. To this DNA-RNAP-peptide 33 mixture, at first 5 μM NTPs without UTP and [α-^32^P] CTP were added to form the EC_23_ complex, which was further chased with 250 uM of all the NTPs and 100 μg/ml rifampicin to ensure single round transcription. The reactions were stopped by adding phenol followed by ethanol precipitation. For the lac promoter, we conducted multiple round transcriptions in the absence of rifampicin using the same reaction conditions. Reaction products were separated using 8% sequencing gel, the image was visualized in a phosphorimager, and band intensities were quantified using ImageQuant 5.2 software.

To assess the effect of peptide 33 on different stages of transcription, it was added at different stages of transcription using the same DNA template as before. (1) Before RNAP: 5 μM peptide 33 was added to 10 nM T7A1-T1T2 template DNA in T buffer for 10 min and then 50 nM RNAP was added and incubated for a further 10 min, following which transcription was initiated with NTPs. (2) After RNAP: here peptide was added after the formation of RNAP-DNA complex at 37 °C, then transcription reaction was initiated. (3) Initiation: in this case, at first an EC_23_ complex was formed, to which the peptide was added, and the complex was chased with all the NTPs. (4) Elongation: in this stage, the peptide was added together with the NTPs used for chasing the EC_23_. The component of the reaction mixtures was the same as before and the transcripts were visualized in the same way as above.

### Microarray analysis

The MG1655 strain was at first transformed with pNL150 plasmids expressing WT Psu, peptide 33, peptide 33CTDΔ8, and the empty vector. Overnight cultures of these strains were subcultured in 10 ml LB with appropriate antibiotics and were allowed to grow until *A*_600_ reached 0.3 to 0.4. The cell pellet was resuspended in 1 ml of RNAlater. RNA isolation and the microarray experiments were performed by Genotypic Technology. Two independent biological replicates for each strain were taken for the analyses. A fold change in gene expression for each strain was calculated relative to the empty vector control. We have considered the genes whose expressions were changed 2-fold (log 2) or more relative to the empty vector control (10). These 2-fold up-regulated and down-regulated datasets were compared with that obtained for the Rho mutants (10, 11, 12, 13, 14) using Venn-diagram tools to find the common and unique genes that are affected by the expressions of the peptides. All the Venn diagrams were prepared using the interactive Venn diagram tool (38). The downregulated genes were analyzed by pathway enrichment analysis by DAVID (39) and network analysis by STRING DB (40) to find the pathways involved with these genes.

### RNA quantification by RT-qPCR

For the RT-qPCR assays, the WT MG1655 strain was transformed with pNL150 plasmid expressing peptide 33, peptide 33Δ8, peptide 33Δ12, peptide 133, and also with the empty vector. After induction with 100 μM IPTG for peptide 33, peptide 33Δ12, peptide 133 and vector, and 50 uM IPTG for peptide 33Δ8, cells were harvested when the *A*_600_ reached ∼0.3 to 0.4. As the cell growth was inhibited in the presence of peptide 33, cell amounts were normalized based on the *A*_600_ values. The total RNA was isolated using the RNeasy Plus Mini Kit (Qiagen), and the complementary DNA (cDNA) was made using random hexamers and SuperScript III Reverse Transcriptase. The amount of cDNA produced during the PCR cycles was monitored in real-time using SYBR green dye in the CFX96 real-time PCR detection system.

For the validation of the microarray results, RT-qPCR reactions were performed for selected genes. In these assays, the threshold cycle (Ct) was calculated from the midpoint of the sigmoidal curve obtained by plotting the fluorescence intensity against the number of PCR cycles. The fold change was calculated using 2^−ΔΔCt^ in the mRNA level in the presence of the peptides, with respect to the empty vector, where C_t_ = the number of threshold cycles; ΔC_t_ = (C_t_ of target gene – C_t_ of internal control); and ΔΔC_t_ = ΔC_t_ in presence of peptides –ΔC_t_ in the presence of an empty vector. The level of *rpoC* mRNA was used as an internal control. We have earlier observed that, unlike the ribosomal RNAs, the rpoC mRNA level does not change upon inhibition of the Rho function. Hence, we used the level of this rpoC RNA as an internal control (10, 12). The primer pairs to amplify the 200 bp product that were designed to correspond to the early middle region of the test genes. Fold change of a single gene at a time was calculated as 2^−ΔCt^ in mRNA levels in the presence of peptides with respect to uninduced peptides, where ΔC_t_ = C_t_ (induced) – C_t_ (uninduced).

Similarly, to see the *in vivo* inhibition of transcription the primer pairs that amplify 200-nt size were designed corresponding to the early-middle region of the test genes (*lacZ, r,* and *osmE* promoters). RNA isolation and cDNA preparation was done as described above. The fold change of a single gene was calculated as 2^−ΔCt^, where ΔC_t_ = C_t_ (induced) – C_t_ (uninduced). The mRNA levels in the presence of peptides were normalized to uninduced peptides.

### Quantification of peptide 33 *in vivo*

For the quantification of peptide 33 *in vivo*, cells were transformed with peptide 33 and pNL150 vector, and total RNA was isolated after induction of cells with 100 μM IPTG. The amount of cDNA produced during the PCR cycles was monitored at 25× cycles of cells expressing peptide 33 and those having only vector. The amount of peptide 33 transcript was quantified using peptide 33 internal primers (RS1878/RS1817). RS1878/1817 primer pair amplifies only in the presence of peptide 33 cDNA.

### Cell morphology by microscopy

To observe the effect of peptides on the cell morphology, we transformed MG1655 cells with pNL150 alone and pNL150 expressing peptide 33 and peptide 33Δ12. From the primary culture, we inoculated 1% secondary culture with two different concentrations of IPTG (0 μM and 50 μM). At various time points, 2 ml of actively growing *E. coli* culture was harvested. The cells were then resuspended in 500 μl of PBS, mixed with 5 ml of chilled methanol, and incubated on ice for 30 min. The cells were centrifuged at 2000 rpm for 10 min, and the pellet was dissolved in 500 μl of chilled 4% paraformaldehyde, followed by a 10-min incubation on ice. After the incubation was over, the cells were collected by centrifugation and resuspended in methanol. A small drop of glycerol (10 μl) was added to the center of the slide, and the coverslip was mounted onto the slide. The cells were then observed and imaged using an LSM900 (Zeiss confocal microscope), and the images were analyzed using ImageJ FIJI software (https://imagej.net/software/fiji/downloads; 41, 42) to calculate the cell length for both the peptide-induced and uninduced cells. The average length of the cells at each time point was plotted using SigmaPlot (Version 15.0).

### Statistical analysis

A two-way ANOVA statistical analysis was performed using SigmaPlot version 15.0 to calculate the variance between data sets. All pairwise multiple comparisons were done by the Holm–Sidak method with a *p*-value threshold of <0.050. All error bars represent the SD of defined experimental replicates. In the figures following symbols were used to indicate the statistical significance of different data: ns = not significant, ∗*p* ≤ 0.05, ∗∗*p* ≤ 0.01, ∗∗∗*p* ≤ 0.001, and ∗∗∗∗*p* ≤ 0.0001 (please see the supplementary data for the details of these analyses). All the bar graphs were represented as simple bar graphs overlaid with scatter plots to represent individual data points of each experiment.

## Data availability

All raw microarray data as well as other data are available upon request to R. S.

## Supporting information

This article contains supporting information.

## Conflict of interest

The authors declare that they have no conflicts of interest with the contents of this article.
