# Supplementary material for: Peptides designed from a bacteriophage capsid protein function as synthetic transcription repressors
Source: J Biol Chem. 2023 Oct 20;299(12):105373. doi: 10.1016/j.jbc.2023.105373 (PMC10692717; doi:10.1016/j.jbc.2023.105373)
Supplement: Supplementary data [file mmc1.pdf]

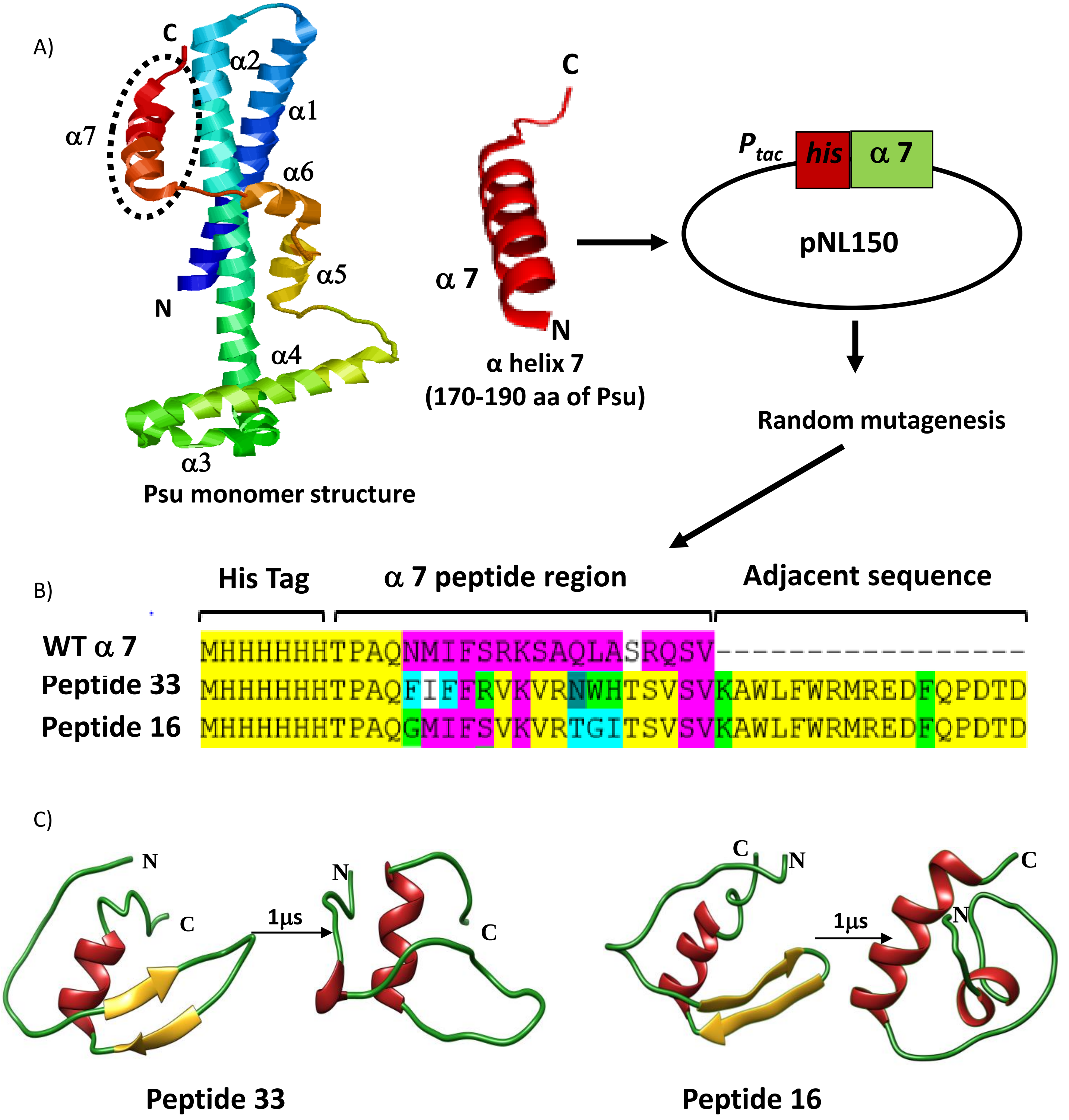

Figure S1: A) Structure of Psu, where helix-7 is depicted [9]. Helix-7 sequence of Psu was cloned in pNL150, and was subjected to random mutagenesis. B) Out of different peptide sequences evolved from the genetic screen of the mutagenized library of helix-7 DNA sequence, peptide 33 and peptide 16 were the most efficient in inhibiting Rho-function [10]. In these peptides a part of the vector sequence were also added to the c-terminal of the mutagenized helix-7 sequence as shown. C) Predicted structures of these two peptides and their dynamic conformational changes as obtained from 1μs MD simulation.

Pathway analyses of Down regulated genes:

Category	Term
KEGG_PATHWAY	eco00630:Glyoxylate and dicarboxylate metabolism
KEGG_PATHWAY	eco01200:Carbon metabolism
KEGG_PATHWAY	eco00052:Galactose metabolism
KEGG_PATHWAY	eco00500:Starch and sucrose metabolism
KEGG_PATHWAY	eco01100:Metabolic pathways
KEGG_PATHWAY	eco02010:ABC transporters
KEGG_PATHWAY	eco01110:Biosynthesis of secondary metabolites
KEGG_PATHWAY	eco02060:Phosphotransferase system (PTS)

- Pathway analysis was performed using DAVID bioinformatics database
- -2 fold and down regulated genes were selected for analysis.
- Top enriched pathways were selected

Figure S2A: The metabolic pathways that the peptides induced >2-fold downregulated genes belong to.

RT-PCR analyses to measure the level of peptide 33 mRNA *in vivo*

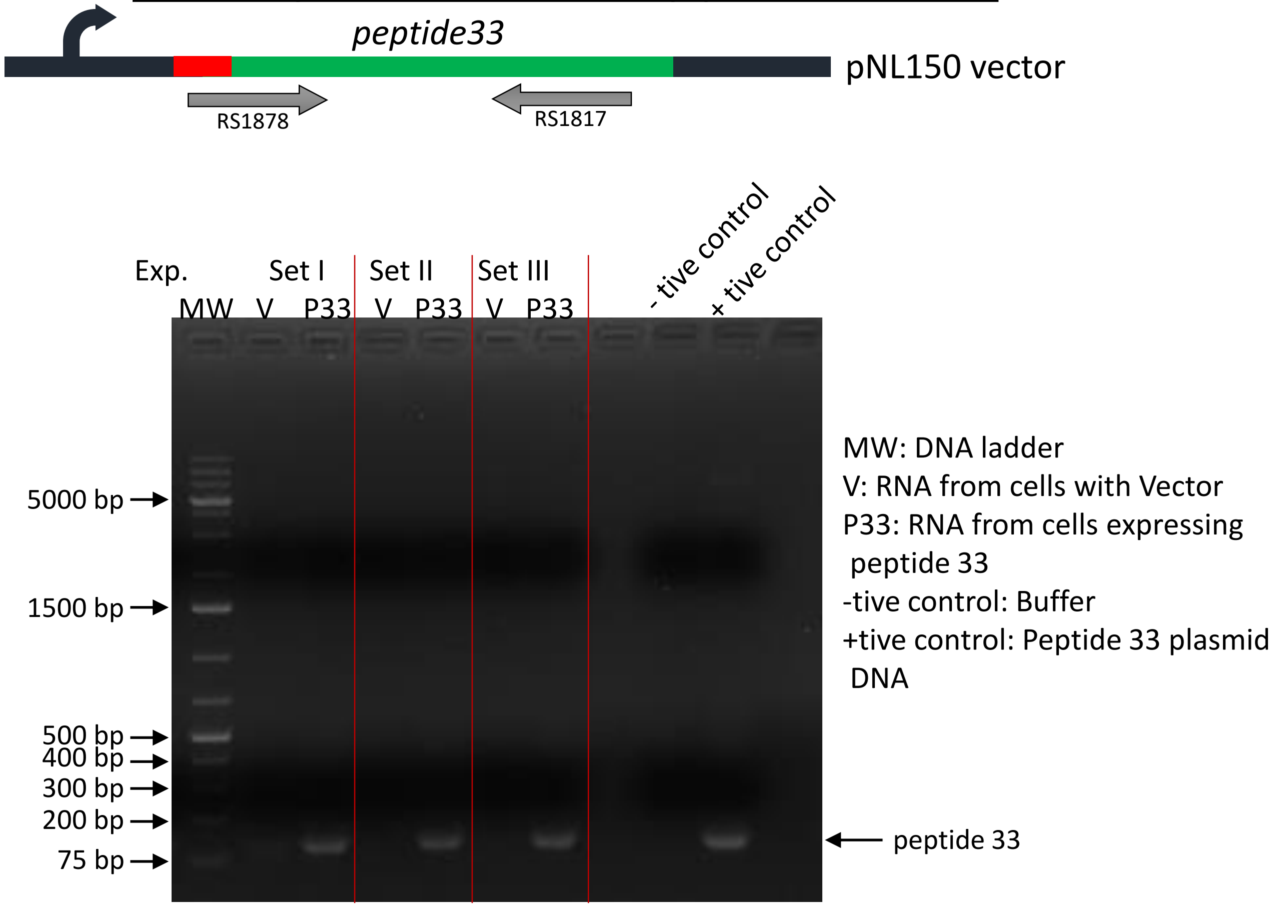

Figure S2B: RT-PCR products (arrow) corresponding to the region of *peptide 33* prepared from the total RNA obtained from MG1655 strains either expressing or not expressing peptide 33.

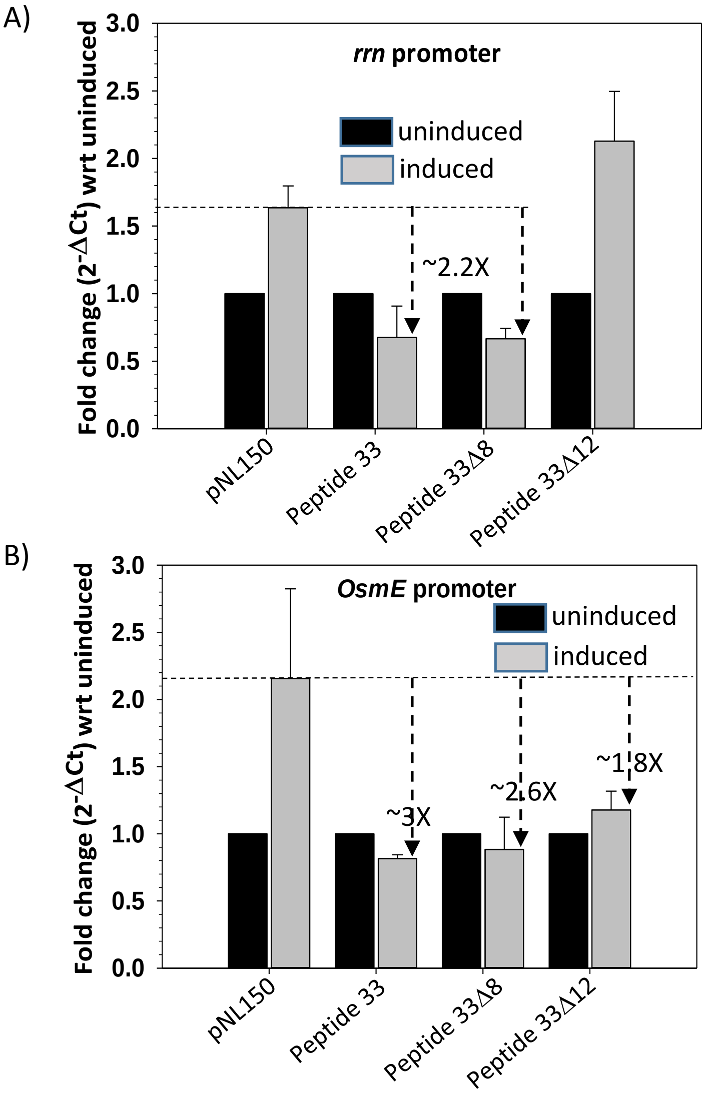

Figure S3: Bar diagrams showing the *in vivo* activities of A) P_{rrnA} and B) P_{osmE} promoters in the presence of the peptides. Fold-change values from the qRT-PCR data were obtained relative to the un-induced ones. Reductions of fold change induced by each peptides relative to the level of that obtained in the presence of the vector pNL150 are indicated.

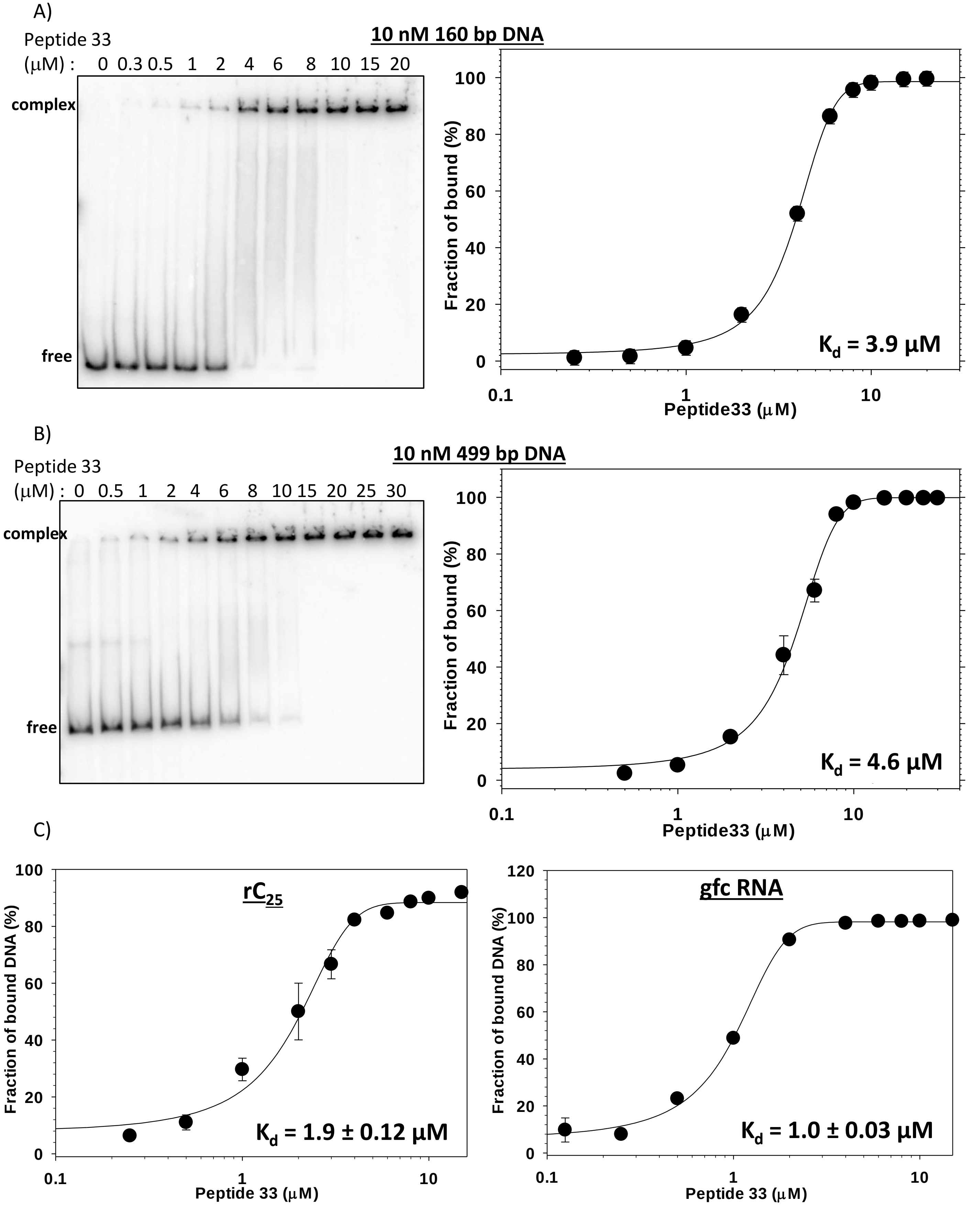

FigureS4. A) Gel-shift assays showing peptide 33 binding with 160bp dsDNA with the binding constant (K_d) of 3.9 μM peptide33 B) Gel-shift assays showing peptide33 binding with 499bp dsDNA with the binding constant (K_d) of 4.6 μM peptide33. C) Binding parameters for peptide33 interaction with RNA (rC(25) and gfc-RNA).

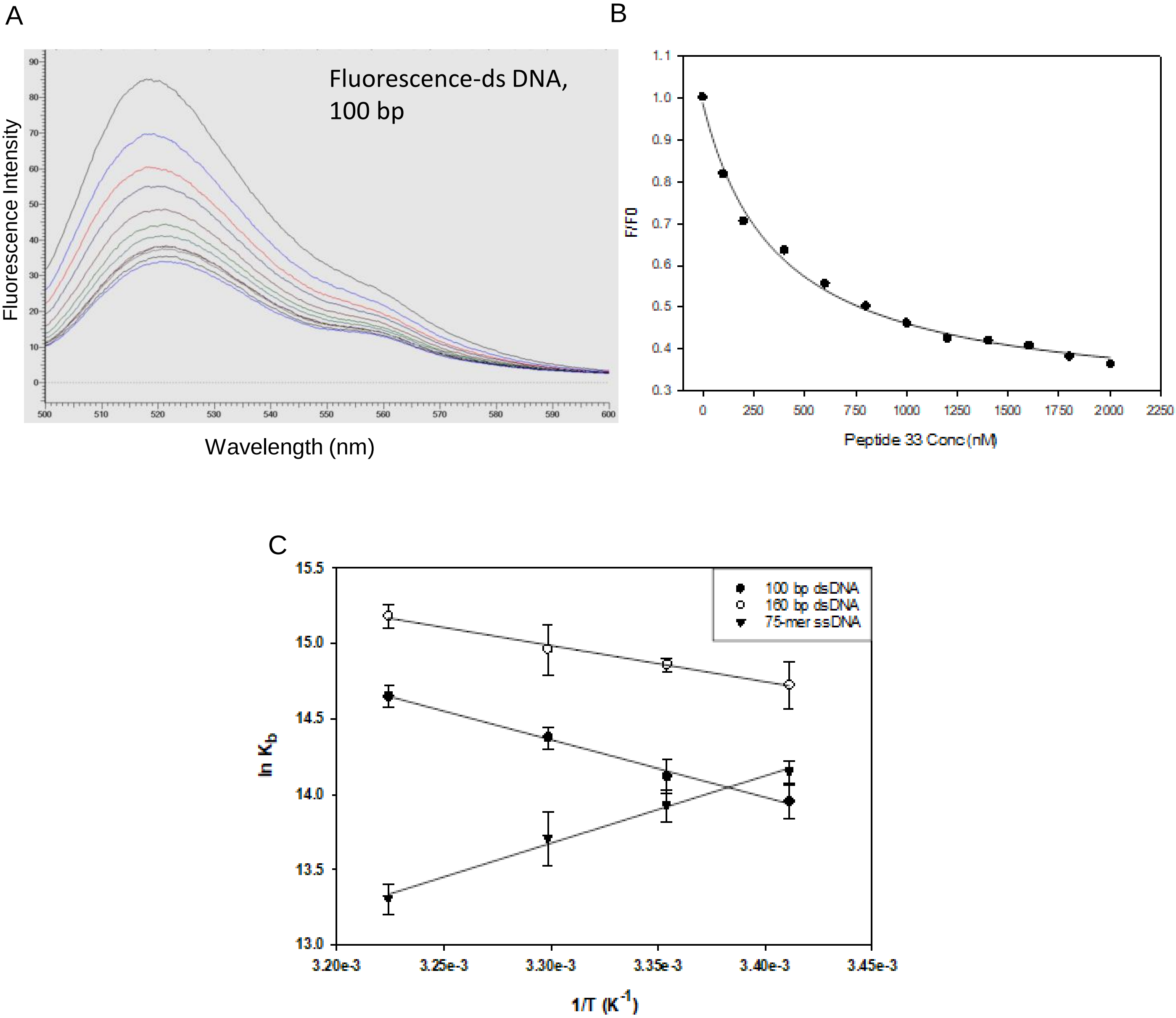

Figure S5: Fluorescence quenching assay for Peptide –DNA interactions: (A) A representative curve showing quenching of fluorescence signal of DNA with a gradual increase in the concentration of peptide 33. (B) Plot showing the change in the fluorescence intensity at 520 nm , normalized to the free DNA (F/F_0), with the addition of peptide 33. The dissociation constant (K_d) was calculated by fitting the curve to the hyperbolic decay model. (C) Van't Hoff plot for the interaction of Peptide 33 with 100 bp and 160 bp dsDNA, and 75-mer ssDNA: The plots of $\ln K_b$ against 1/T show a linear relationship with an $r^2 > 0.99$. Each point represents the average value of three individual experiments. Error bars represent SEM.

Global impact of peptide on pathways:

Category	Term
KEGG_PATHWAY	eco02040:Flagellar assembly
KEGG_PATHWAY	eco02030:Bacterial chemotaxis
KEGG_PATHWAY	eco02020:Two-component system
KEGG_PATHWAY	eco00543:Exopolysaccharide biosynthesis
KEGG_PATHWAY	eco00440:Phosphonate and phosphinate metabolism
KEGG_PATHWAY	eco00730:Thiamine metabolism
KEGG_PATHWAY	eco00051:Fructose and mannose metabolism

- Pathway analysis was performed using DAVID bioinformatics database
- 2 fold and up regulated genes were selected for analysis.
- Top enriched pathways were selected

Figure S6: The global impact of peptide on pathways of *Escherichia coli* MG1655

Table S1: Oligos used in the study.

Name	description	sequence
RS58	Forward primer binding upstream of promoter sequence of pTL61T plasmid	ATAAACTGCCAGGAATTGGGGATC
RS-RK1	Reverse primer binding on lacZ sequence of pTL61T plasmid	GTTTTCCCAGTCACGAC
RS139	FP to make template for λtr1 RNA synthesis	TTAATACGACTCACTATAGGGAGATCGAGAGGGACA CGGGCG
RS209	primer binding to rutA site of trpT’	GGAAAAATGACGTAAGTTGAC
RS224	34 mer polydC for primary site binding of rho	CCCCCCCCCCCCCCCCCCCCCCCCCCCCCCCCCC
RS256	5' Fluoreciene labelled RS224	CCCCCCCCCCCCCCCCCCCCCCCCCCCCCCCCCC
RS258	5' Fluoreciene labelled RS58	ATAAACTGCCAGGAATTGGGGATC
RS341	RP to make template for λtr1 RNA synthesis	GAATTGTGAGCGCTCACAATTCGGATCCTTAGATAACAA TTGATTGAATG
RS347	Reverse primer to amplify 292bp dsDNA on RS102 plasmid	CAGCACACATCGCCTGAAAGACTAGTCAGGATGATGGTA TTGATTTACTGGTGTTATGTTGCGG
RS590	5' Fluoreciene labelled 30mer oligo	AAGCTTCGCCCGTGTCCCTCTCGATCTCCC
RS952	Ptac Promoter sequence, before MCS (for sequencing)	TGACAATTAATCATCGGCTCGTATAATGT
RS1141	r(C)25-RNA OLIGO	CCCCCCCCCCCCCCCCCCCCCCCCC
RS1230	pNL150 vector specific Reverse Primer, 100bp downstream of gene	AGTTCGGCATGGGGTCAGGTGGGACC
RS1466	qRT FP primers for dppA gene	TTTGATGGCTACTGGGGCAC
RS1467	qRT RP primers for dppA gene	AGATAACCGACGTTCAGCCC
RS1468	qRT FP primers for dppB gene	TTCGATCACACAGCGGTTGG
RS1469	qRT RP primers for dppB gene	AGATGGCGGTGTCGATTAGC
RS1816	26 mer ssDNA oligo used for binding assays	ATCTTCTCTCATCCGCCAAAACAGCC
RS2037	gfcAp FP for RNA template synthesis	TAATACGACTCACTATAGGGAGAGCTGCTTGTTTAA AGCTGACTGG
RS2038	gfcAp RP for RNA template synthesis	CTATCCCCCTCTGGAAATACGAC
RS2117	qRT FP primers for tnaB gene	TATTTACGGGATCGGCGGTG
RS2118	qRT RP primers for tnaB gene	ATAAGATTGCCGCCCCAGAC
RS2119	qRT FP primers for tnaA gene	CCGCCAAGAAAGATGCGATG
RS2120	qRT RP primers for tnaA gene	CCGTCATACAGACCTACCGC
RS2123	qRT FP primers for ydeN gene	TGGGGTTATGCGGGTAATCG
RS2124	qRT RP primers for ydeN gene	TACCCGACAGCTCTTGATGC

RS2127	qRT FP primers for gatD gene	TGTTTGCATCGCACCGAAAG
RS2128	qRT RP primers for gatD gene	AGCCGATTACCGTTGGTCTG
RS2129	qRT FP primers for gutM gene	CCGTGCCTTCGACACACTAT
RS2130	qRT RP primers for gutM gene	TGGGGAAAGATCACATCGGG
RS2131	qRT FP primers for katE gene	TTGCTGAAAACGAACAGGCG
RS2132	qRT RP primers for katE gene	GTTCGGTTCGTAATTCGCCG
RS2135	qRT FP primers for gatC gene	ACCGCCCTGATCCATTGAAG
RS2136	qRT RP primers for gatC gene	TCGTGGAAATCTGTTCCGCA
RS2161	qRT FP primers for tnaC gene	AATTGTCGATCACCGCCCTT
RS2162	qRT RP primers for tnaC gene	TAACACGAATGCGGAACGGT
RS2230	75 mer ssDNA oligo used for binding assays	TGATCCTCCTCCTCCTGATCCTCCTCCTCCTGATCCT CCTCCTCCTGAGCGTTTCATCATTTCGAAGAAATCGTC
RS2381	Reverse primer for amplification of 100 bp DNA with pRS106	GCTGTAAGTATCCTATAGG
RS1817	Forward primer for amplification of peptide33	TCTCATCCGCCAAAACAGCCAAGC
RS1878	Reverse primer for amplification of peptide33	CATCATCATCATACCCCTGCGCAGTTCATATTTTTTCGCG
